# The True Host/s of Picobirnaviruses

**DOI:** 10.3389/fvets.2020.615293

**Published:** 2021-01-20

**Authors:** Souvik Ghosh, Yashpal S. Malik

**Affiliations:** ^1^Department of Biomedical Sciences, Ross University School of Veterinary Medicine, Basseterre, Saint Kitts and Nevis; ^2^College of Animal Biotechnology, Guru Angad Dev Veterinary and Animal Science University, Ludhiana, India

**Keywords:** picobirnavirus, true host/s, opportunistic animal pathogen, prokaryotic virus, fungal virus

## Abstract

Picobirnaviruses (PBVs) are bisegmented double-stranded RNA viruses that have been detected in a wide variety of animal species including invertebrates and in environmental samples. Since PBVs are ubiquitous in feces/gut contents of humans and other animals with or without diarrhea, they were considered as opportunistic enteric pathogens of mammals and avian species. However, the virus remains to be propagated in animal cell cultures, or in gnotobiotic animals. Recently, the classically defined prokaryotic motif, the ribosomal binding site sequence, has been identified upstream of putative open reading frame/s in PBV and PBV-like sequences from humans, various animals, and environmental samples, suggesting that PBVs might be prokaryotic viruses. On the other hand, based on the detection of some novel PBV-like RNA-dependent RNA polymerase sequences that use the alternative mitochondrial genetic code (that of mold or invertebrates) for translation, and principal component analysis of codon usage bias for these sequences, it has been proposed that PBVs might be fungal viruses with a lifestyle reminiscent of mitoviruses. These contradicting observations warrant further studies to ascertain the true host/s of PBVs, which still remains controversial. In this minireview, we have focused on the various findings that have raised a debate on the true host/s of PBVs.

## Introduction

Picobirnaviruses (PBVs) are bisegmented double-stranded RNA viruses that belong to the sole genus *Picobirnavirus* within the family *Picobiraviridae* (1). Picobirnaviruses have been widely reported in fecal samples/gut contents of humans and various animal species with, or without diarrhea (1–3). Traditionally, PBVs are considered as opportunistic enteric pathogens of mammals and avian species (1–5). On the other hand, PBVs have also been detected in invertebrates and environmental samples (6–9). During the past few years, the whole genomes, or at least the complete/nearly complete gene segment-2 sequences of several PBV strains from humans, different animal species and environmental samples have been obtained using next generation sequencing technologies, or a modified non-specific primer-based amplification method (1, 4, 6–8, 10–20). Analyses of the expanded repertoire of diverse full-length/nearly full-length PBV sequences revealed remarkable features in the PBV genome including those that suggest that PBVs might be actually prokaryotic or fungal viruses (1, 4, 6–8, 10–21). In this minireview, we have discussed the various findings that have raised a debate on the true hosts of PBVs.

## Picobirnavirus Morphology and Genome

**Figure 1:**
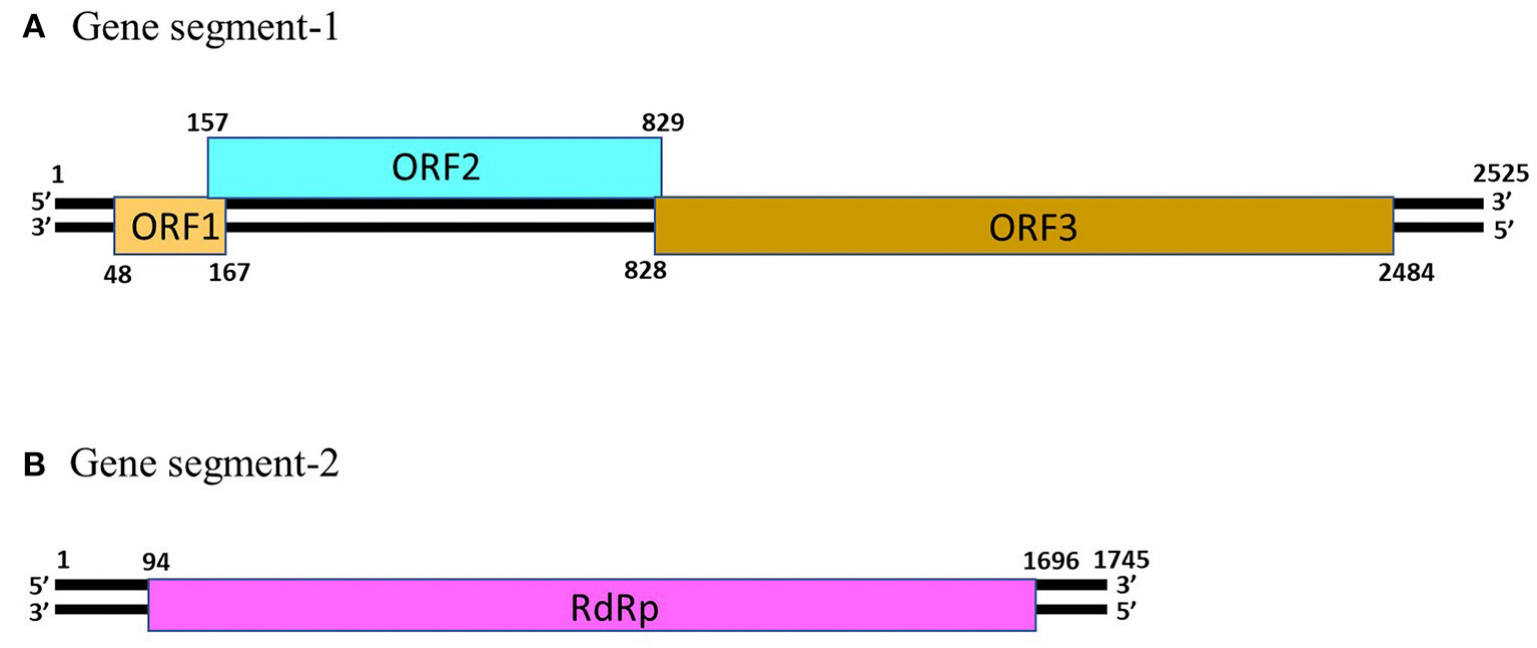
The genomic organization of human picobirnavirus genogroup-I strain Hy005102. **(A)** Gene segment-1 (GenBank accession number AB186897) of PBV strain Hy005102 consists of 3 putative open reading frames (ORF), designated as ORF1, ORF2 and ORF3. The ORF3 codes for a precursor of the viral capsid protein. **(B)** Gene segment-2 (GenBank accession number AB186898) of PBV strain Hy005102 possess a single ORF that encodes the viral RNA-dependent RNA polymerase (RdRp).

Picobirnaviruses are spherical, non-enveloped viruses with a diameter of ~33–37 nm (1). Since PBVs are non-cultivable, information on the virus capsid is based on those of recombinant virion-like particles (22). PBV possess a simple icosahedral capsid that is composed of 60 symmetric dimers (22). The PBV capsid contains two segments of dsRNA, designated as gene segment-1 (~2.2–2.7 kb in size) and −2 (~1.2–1.9 Kb in size) (Figure 1) (1–3, 5). Because of their small size (“*pico*” in Spanish) and bi-segmented (“*bi*” in Latin) nature of the viral genome, the viruses were named “Picobirnaviruses” (23). However, PBVs with monopartite genomes have also been reported in a few studies (6, 24, 25).

The gene segment-1 of PBVs consists of 2 or 3 open reading frames (ORFs), designated as ORF1, ORF2, and ORF3 from the 5′- end (Figure 1) (1, 13). The ORF3 codes for a precursor of the capsid protein which undergoes autocatalytic cleavage to generate the mature capsid protein, and a charged peptide that is believed to interact electrostatically with the viral RNA (22). The ORF2 encodes a protein characterized by repeats of the ExxRxNxxxE motif (1, 26). However, the function/s of this protein is not yet known. On the other hand, the functionality, or even the presence of ORF1 in gene segment-1 of PBVs remain to be elucidated (1, 13, 26). The PBV gene segment-2 possess a single large ORF that encodes the RNA-dependent RNA polymerase (RdRp) (Figure 1) (1–3, 5). The 5′- (GUAAA) and 3′- (ACUGC) termini sequences appear to be conserved in the gene segment 2 of PBVs (1, 10, 14–20). The PBV RdRp catalyzes RNA synthesis with both single-stranded RNA and dsRNA templates, and transcription occurs in a semi-conservation manner (27). During encapsidation, the RdRp is believed to form a complex with the viral genome (27).

**Figure 2:**
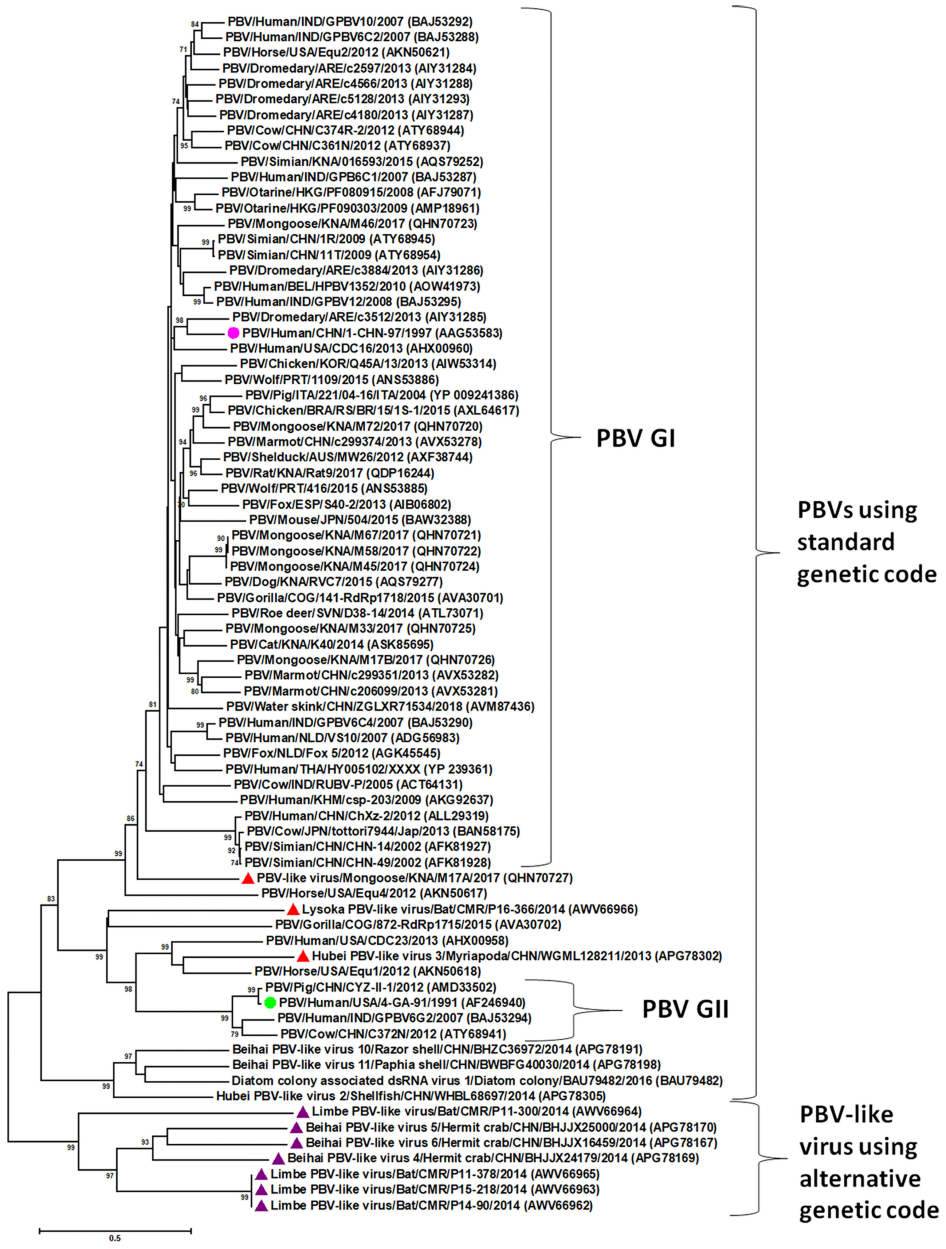
Phylogenetic analysis of the picobirnavirus (PBV) and PBV-like RNA-dependent RNA polymerase (RdRp) sequences. The phylogenetic tree was constructed by the Maximum Likelihood method using the MEGA6 software. Phylogenetic distances were measured using the LG + G model of substitution. The tree was statistically supported by bootstrapping with 500 replicates. Bootstrap values <70% are not shown. Scale bar, 0.5 substitutions per amino acid. The name of the PBV strain includes virus/host of detection/country/common name/date of collection. GenBank accession numbers are shown in parentheses. Pink circle: prototype PBV genogroup-I (GI) strain; green circle: prototype PBV GII strain; purple triangles: PBV-like viruses that use an alternative mitochondrial genetic code to translate the RdRp and cluster separately from PBVs using the standard genetic code; red triangles: PBV-like strains that use an alternative mitochondrial genetic code to translate the RdRp, yet cluster within PBVs using standard genetic code.

Picobirnaviruses exhibit high genetic diversity within and between host species (1–5, 10–18, 24, 28–30). A viral metagenomics study in diarrheic free-ranging wolves has provided evidence for genetic reassortment events among PBVs (31). Most studies on genetic diversity of PBVs are based on gene segment-2/RdRp sequences (2–5). The phylogenetic analysis of PBV RdRp sequences has been shown in Figure 2. Majority of the PBV RdRp sequences reported so far use the standard genetic code for translation, whilst, recently, some novel PBV-like RdRp gene sequences that use an alternative mitochondrial genetic code have been detected in bats, humans, invertebrates, and a mongoose (6, 11, 12, 14). Picobirnaviruses using the standard genetic code cluster separately from PBVs using the alternative mitochondrial genetic code (12, 14). However, three PBV-like RdRp sequences (from a bat, a mongoose and a myriapod) that use an alternative mitochondrial genetic code were found to cluster with PBVs using the standard genetic code (12, 14). These unique PBV-like RdRp sequences have been discussed in the section “evidence that picobirnaviruses might infect fungi” of the review article. Within the cluster of PBV RdRp sequences using the standard genetic code, PBVs are further classified into two genogroups (genogroup-I (GI) and GII), although several PBVs that could not be classified into either genogroup have also been reported (1–5, 10–20, 24, 29, 30). Picobirnavirus GI strains have been more frequently detected compared to GII strains (2, 3, 5).

## Picobirnavirus Infection in Humans and Animals

Picobirnavirus infection in humans and animals have been excellently reviewed by Ganesh et al. (2) and Malik et al. (3). Picobirnaviruses have been detected in sporadic cases of diarrhea as well as associated with outbreaks of gastroenteritis in humans and in a wide variety of animals (1–3, 5). They are often reported in coinfection with other enteric pathogens (1–5, 32–34). On the other hand, PBVs have also been frequently detected in apparently healthy humans and animals without diarrhea (1–3, 5, 14, 15, 18).

Based on studies in immunocompromised and immunosuppressed humans, PBVs were considered as opportunistic enteric pathogens (2, 3). PBVs have been reported more frequently in HIV-infected patients with diarrhea than those without diarrhea (35–38), and in fecal samples from kidney transplant patients (39, 40). In organ transplant recipients, PBVs were predictive of the occurrence of severe enteric graft-vs.-host disease (GVHD), and correlated with the high levels of GVHD severity markers in feces (41). Recently, it was demonstrated that pregnant women with type 1 diabetes (T1D) are more likely to harbor PBVs than those without T1D (42).

Prolonged fecal shedding of PBVs, characterized by alternating periods of high-, low-, and no- virus detection, have been observed in asymptomatic animals, and in healthy and HIV-infected humans (35, 38, 43–50). Various factors, such as age, stress, physiological status and environmental conditions have been proposed to influence the PBV shedding patterns in infected humans and animals. In studies monitoring PBV shedding patterns in animals, highest excretion rates were observed during the lactogenic period in pigs and sheep, whilst increased viral shedding occurred at a young age in rabbits (weaned), broilers (aged 2–7 weeks) and rhea (~3 weeks of age) (44, 46, 49, 51, 52). Based on these observations, it has been suggested that animals could acquire PBV infection early in life, followed by establishment of persistent infection, with infected adults serving as asymptomatic carriers (2, 3, 5).

Although mostly reported in feces/gut contents, PBVs have also been detected, albeit rarely, in the respiratory tract of humans (from individuals with unexplained respiratory disease in Netherlands and cases of severe acute respiratory infection in Uganda) and animals (asymptomatic cattle, monkeys and pigs) and in the plasma of a febrile horse, suggesting an expanded tissue tropism of the virus (10, 24, 53–55).

PBVs exhibit high genetic diversity between and within host species, and phylogenetically, species specific clustering patterns have not been observed so far (1, 4, 10–18, 24, 28–31). Nevertheless, based on sequence identities and phylogenetic analysis, interspecies transmission events including zoonoses have been proposed for PBVs, although several of these studies were based on partial gene segment-2 sequences and are not conclusive (2–5, 11, 40, 43, 53, 56–60).

## Evidence that Picobirnaviruses Might Infect Prokaryotes

One of the intriguing recent findings on PBVs has been the identification of a classically defined prokaryotic motif, the ribosomal binding site (RBS) sequence (also known as Shine-Dalgarno sequence), upstream of putative ORF/s in PBV gene segment-1 and−2 sequences (Table 1) (13, 21). In prokaryotes, the RBS sequence (AGGAGG), or its subset (4-, 5-, or 6- mer of AGGAGG) enables the mRNA to bind to ribosome, resulting in initiation of translation, and is mostly located anywhere between −18 and −4 nucleotides upstream from the start codon (21, 61–63). Certain viruses that infect prokaryotes have been shown to be highly enriched for RBS sequences, such as the bacteriophages with segmented dsRNA genomes of family *Cystoviridae* (13, 21, 64, 65). Similar to prokaryotic mRNAs and the cystoviruses, the RBS sequence, or its subset has been found to be conserved upstream of putative ORF/s (putative ORF1, 2 and 3 in gene segment-1 and putative ORF for RdRp in gene segment-2) in published PBV sequences from humans, animals and environmental samples (4, 7, 8, 11–18, 21). The presence of the conserved prokaryotic RBS sequence upstream of the putative start codon/s in representative PBV gene segment-1 sequences, gene segment-2 sequences of PBV GI and GII strains, and PBV-like RdRp sequences are shown in Table 1. In fact, PBVs exhibited an enrichment level for RBS sequences that was higher than those observed in any known prokaryotic viral family (21). Based on these observations, it has been proposed that PBVs might be actually prokaryotic viruses (13, 21). Since not all prokaryotic viruses appear to retain the prokaryotic RBS sequence, and not every bacterial phylum exhibits a high level of enrichment for RBS sequences, it has been hypothesized that viruses enriched for RBS sequences, such as PBVs, were more likely to infect bacteria that highly conserves the RBS sequences for its own genes (21).

Table 1The location of the prokaryotic ribosomal binding site (RBS) sequence upstream of the putative open reading frame/s (ORF) in picobirnavirus (PBV) and PBV-like sequences.

**Table:** 

**Host/PBV strain**	**PBV gene segment-1**			
	**GenBank accession number**	**5^**′**^- UTR of putative ORF-1**	**5^**′**^ -UTR of putative ORF-2**	**5^**′**^ -UTR of putative ORF-3 (ORF3 codes for the Capsid)**
**(A) PBV GENE SEGMENT-1**				
Human/Hy005102	AB186897	GAAGGAGAGATGTT**ATG**AA	AAAGGAGGTTATTTA**ATG**AC	GCAGGAGGTTTATC**ATG**AA
Pig/221/04-16/ITA/2004	KF861770	AAAGGAGAATGATCTAAC**ATG**AA	TAAGGAGGTGAAAGTT**ATG**CT	ATGGAGGCTAAT**ATG**AA
Otarine/PF090307	KU729753	AAAGGAGATGTGCATTTTTA**ATG**GA	AAAGGAGGAAATGT**ATG**AC	AAAGGAGTGTTTAAT**ATG**TC
Roe deer/SLO/D38-14/2014	NC_040752	GAAGGAGGAG**ATG**CT	AAAGGAGGACACAGTTTC**ATG**AC	TAAGGAGAATATTACAA**ATG**AA
Wolf/PRT/891/2015	KT934310	AAAGGAGGAACTT**ATG**TGTAGTAA	AAAGGAGGA**ATG**CA	AAAGGAGACCATTAATA**ATG**GC
Marmot/c130145_g1_i1_libraryA_2712	KY928752	AAAGGAGGACTGTTGGA**ATG**TT	AAAGGAGGTC**ATG**TA	AAAGGATTTATTATC**ATG**AG
Rabbit/ R5-9	NC_038919	ATAGGAGGAAGTGCTTATGAAAC**ATG**CA	AAAGGAGGTGTCAATGGT**ATG**AC	CTAGGAGGTAAAAT**ATG**AA
Macaque/WUSTL	MG010886	AAAGGAGAACAGATC**ATG**GT	AAAGGAGGAAACGTTT**ATG**AC	CTGGAGAAAATT**ATG**AG
Chicken/ChPBV-S1-ctg289/2013-HUN	MH425579	AAAGGAGGAATCAGTGTTTT**ATG**GA	AAAGGAGGTATATA**ATG**AC	ATAGGAGGAATAAAT**ATG**AA
Turkey/USA/MN-1/2011	KJ495689	AAAGGAGGCGTACGTA**ATG**GT	CAAGAAAGGAAGTGACAAAC**ATG**AC	TTGGAGGAATTATCG**ATG**GG

**Table:** 

**Host/Genogroup/PBV strain**	**PBV gene segment-2**		**Host/Genogroup/PBV strain**	**PBV gene segment-2**	
	**GenBank accession number**	**5′-UTR of putative ORF(ORF codes for the RdRp)**		**GenBank accession number**	**5′** **-UTR of putative ORF (ORF codes for the RdRp)**
**(B) PBV GENE SEGMENT-2**					
Human/GI/Hy005102	AB186898	AAAGGAGGACTACTT**ATG**CA	Marmot/GI/c299351	KY928717	AAAGGAGGTTCACGTT**ATG**CC
Pig/GI/221/04-16/ITA/2004	KF861773	AAAGGAGGCTAAGCATT**ATG**CC	Rat/GI/Rat9	MH412924	AAAGGAGGCTTTTCTAT**ATG**CC
Otarine/GI/PF090307	KU729767	AAAGGAGGCCATTACA**ATG**CC	Shelduck/GI/MW26	MH453875	AAAGGAGGCTATCTTT**ATG**CC
Roe deer/GI/SLO/D38-14/2014	NC_040753	AAAGGAGGTTATCGTT**ATG**CC	Chicken/GI/ChPBV-S2-ctg1042/2013-HUN	MH425584	AAAGGAGGTAATGCTT**ATG**CT
Dog/GI/RVC7	KY399057	AAAGGAGGTTCACATT**ATG**CC	Turkey/GI/USA/MN-1/2011	KJ495690	AAAGGAGGTCATTCATGT**ATG**AA
Cat/GI/K40	MF071281	AAAGGAGGTCGCGTA**ATG**CC	Human/GII/4-GA-91	AF246940	AAAGGAGGTTTACT**ATG**AA
Cow/GI/RUBV-P	GQ221268	AAAGGAGGACTACAAA**ATG**TC	Cow/GII/C372N	KY120178	AAAGGAGGTTTACT**ATG**AA
Horse/GI/Equ2	KR902505	AAAGGAGGTTACGTT**ATG**CC	Pig/GII/CYZ-II-1	KP984805	AAAGGAGGTTTACT**ATG**AA
Water skink/GI/ZGLXR71534	MG600064	AAAGGAGGACATTAGAT**ATG**TC	*Mongoose/ND/M17A*	MN563302	TCAGGAGGTTAGTTTCTT**GTG**AT
Wolf/GI/PRT/1109/2015	KT934308	AAAGGAGGTCCGTT**ATG**CC	*Myriapoda/ND/WGML128211*	KX884187	AAAGGAGTTTTACT**ATG**AG
Simian/GI/016593	KY053143	AAAGGAGGCCATCATT**ATG**CC	*Bat/ND/P15-218*	MG693102	AAAGGAGGAAACAAGA**ATG**CC
Mongoose/GI/M17B	MN563301	AAAGGAGGTTCACGTT**ATG**CC	*Hermit crab/ND/BHJJX25000*	KX884060	AGAGAGGGATATCTA**ATG**AA

*The RBS sequence is underlined, whilst the putative start codon is shown with bold font, respectively. The PBV-like sequences that use an alternative mitochondrial genetic code for translating the putative RNA-dependent RNA polymerase (RdRp) are shown with italics. UTR, untranslated region*.

Supporting the hypothesis on prokaryotic hosts, PBVs remain to be successfully propagated in eukaryotic cell cultures (1). However, previous experiences with adapting enteric viruses, especially noroviruses to cell culture have been extremely challenging (66). Furthermore, the lack of a cell culture platform in itself does not rule out the possibility that PBVs are animal viruses. Recently, attempts were made to cultivate PBVs in prokaryotic cells by inoculating 3% cloacal suspension from a PBV positive chicken into brain heart infusion broth (13). The *in vitro* cultures were maintained under aerobic and anaerobic conditions for 2 weeks, and regularly monitored for PBV RNA by RT-qPCR assay. There was no evidence for amplification of PBVs during the study period. Since the sample was collected and frozen long-term to analyze viral nucleic acid rather than maintain bacterial diversity or retain viral infectivity, it might have been possible that the number of cultivable, intact bacteria as well as the low initial infective particles were significantly decreased. However, in the same study, expression analysis in *Escherichia coli* using 6xHis-tagged recombinant PBV segment-1 and western blot assay revealed the *in vivo* functionality of PBV segment-1 sequences containing the RBS motif in a bacterial system (13). The ubiquitous nature of PBVs, especially high prevalence of the virus in environmental samples including sewage water and detection of viral RNA in feces/gut contents from a wide variety of animal species including atypical hosts such as reptiles and invertebrates, and persistent fecal shedding by asymptomatic animals, suggest that PBVs might be prokaryotic viruses of the gut microbiome (1–3, 6–9, 43–52).

On the other hand, certain observations with PBVs are reminiscent of eukaryotic viruses, such as (i) viremia in a sick horse and respiratory tract infections in cattle, humans, monkeys and pigs, (ii) immune response in a rabbit that was temporally associated with PBV excretion, and (iii) autoproteolytic processing of the PBV capsid protein and liposome-perforating capacity of viral particles (10, 22, 24, 53–55, 67). However, similar findings have also been reported for bacteriophages:(i) bacteriophages have been detected in blood (phagemia) and respiratory samples, (ii) immune responses have been raised against prokaryotic viruses, and (iii) autoproteolytic capacities have been demonstrated for bacteriophages (68–72).

## Evidence That Picobirnaviruses Might Infect Fungi

The recent detection of unique PBV-like sequences that lack a putative ORF for RdRp using the standard genetic code, but use an alternative mitochondrial genetic code for translation has further complicated the ongoing debate on true hosts of PBVs (6, 11, 12, 14). These PBV RdRp-like sequences have been detected in bats, humans, invertebrates (crustaceans and myriapods), and a mongoose (6, 11, 12, 14). The human PBV-like RdRp sequences were closely related (99% sequence identities) to that of a bat PBV-like RdRp sequence detected in the same region (11). The PBV-like RdRp sequences were found to translate the putative RdRp using the invertebrate mitochondrial genetic code (transl_table=5, NCBI genetic codes, www.ncbi.nlm.nih.gov/Taxonomy/Utils/wprintgc.cgi#SG5) as well as the mold mitochondrial genetic code (transl_table=4, NCBI genetic codes, www.ncbi.nlm.nih.gov/Taxonomy/Utils/wprintgc.cgi#SG4) (6, 11, 12, 14). Based on phylogenetic analysis of the PBV- and PBV-like RdRp sequences, except for three PBV-like strains (Lysoka PBV-like virus/Bat/CMR/P16-366/2014, Hubei PBV-like virus 3/Myriapoda/CHN/WGML128211/2013, and PBV-like virus/Mongoose/KNA/M17A/2017), the PBV-like RdRp sequences formed a separate cluster that was distinct from the PBVs using standard genetic code including PBV GI and GII strains (Figure 2) (6, 11, 12, 14). The metagenomics data pool reporting these PBV-like RdRp sequences did not reveal any PBV-like capsid sequences (6, 12). Based on these observations, it has been proposed that the PBV-like strains using the alternative mitochondrial genetic code might have a lifestyle that is reminiscent of mitoviruses (12).

Mitoviruses (genus *Mitovirus*, family *Narnaviridae*) are plus-stranded RNA virus-like elements that replicate in the fungal mitochondria, although they have also been reported in plants and invertebrates (thought to be derived from fungal symbionts) (73–75). Mitoviruses lack a capsid, and the viral genome consists of a single long ORF that encodes a deduced protein with the conserved motifs of a viral RdRp. Principal component analysis of the codon usage bias revealed close clustering of the PBV-like RdRp sequences from bats and invertebrates with those of mitoviruses using the mitochondrial genetic code, corroborating the hypothesis that PBVs might behave like mitoviruses (12). By phylogenetic analysis of RdRp sequences from different virus families, PBVs and partitiviruses (known to infect fungi and plants, and share similarities in capsid architecture and genome organization with PBVs) were found to constitute the partitivirus-picobirnavirus clade, which, interestingly, also consisted of some naked RNA replicons that reproduce in algae mitochondria or chloroplasts, translate using a mitochondrial genetic code, and exhibit mitovirus-like behavior (76). Taken together, these findings suggested that PBVs might be fungal viruses.

On the other hand, at least three PBV-like RdRp sequences (P16-366, WGML128211, and M17A from a bat, myriapod and mongoose, respectively) that use the alternative mitochondrial genetic code (that of mold or invertebrate) for translation were found to cluster within PBVs using the standard genetic code (Figure 2) (6, 12, 14). Furthermore, a capsid sequence was identified for the bat PBV-like strain P16-366 (12). Based on phylogenetic analysis of RdRp sequences, it has been hypothesized that the dsRNA viruses of the partitivirus-picobirnavirus clade might have evolved through reassortment events involving gene segments encoding, respectively, a dsRNA virus capsid protein related to those of the major clade of dsRNA viruses (cystoviruses, totiviruses, and reoviruses) and a positive sense RNA virus RdRp (possibly from a naked RNA replicon within the partitivirus-picobirnavirus clade) (76), which might offer a possible explanation for the origin of P16-366. Although the presence or absence of a capsid sequence could not be determined for the mongoose PBV-like strain M17A, the RdRp sequence of M17A retained the various features (5′- terminal nucleotide sequence and the three motifs (DFXKFD, SGSGGT and GDD) in putative RdRp) that are conserved in gene segment-2 of PBVs, and phylogenetically, clustered near PBV GI strains (14). Further analyses are required to decipher the complex evolution of these PBV-like strains.

## Limitations and Future Scope of Research

Studies so far could not establish a consistent association between PBV detection and diarrhea in humans and animals (1, 5). Moreover, there are a few reports on detection of PBVs in respiratory samples and in a serum sample from mammals (10, 24, 53–55). To date, PBVs remain to be successfully propagated in mammalian cell culture systems and/or gnotobiotic animal models (1). As a result, the tissue tropism and pathogenesis of PBVs in mammals remain to be elucidated so far, necessitating further research toward establishing mammalian cell culture systems, intestinal organoids, and/or gnotobiotic animal models that would support the propagation of the virus. On the other hand, the recent speculations that PBVs might actually infect bacteria or fungi were based on analyses of PBV and PBV-like sequences (7, 8, 12–14, 21, 76). To date, PBVs have not yet been isolated from cultured bacterial or fungal cells. In order to conclusively establish that PBVs are prokaryotic or mycoviruses, future research should focus on successful propagation of PBVs in various prokaryotic and fungal cell culture systems, especially those derived from the gut microbiome of mammals.

## Conclusions

Since PBVs have been mostly detected in feces/gut contents of animals and humans with or without diarrhea, they were considered as opportunistic enteric pathogens of mammals (2, 3). However, the identification of the prokaryotic RBS sequence upstream of putative ORF/s in PBV and PBV-like sequences indicate that PBVs might actually infect bacteria (7, 8, 13, 21). Furthermore, by phylogenetic analysis of viral RdRp sequences, the partitivirus-picobirnavirus clade has been hypothesized to have originated in an as-yet-undiscovered lineage of prokaryotic RNA viruses (76). On the other hand, detection of some novel PBV-like RdRp sequences that use the alternative mitochondrial genetic code (that of mold or invertebrates) for translation has raised the speculation that PBVs might be fungal viruses with a mitovirus-like lifestyle (6, 12, 14). Based on these observations, it might be possible that PBVs actually infect the gut microbiome of mammals and not mammalian cells. Taken together, these contradicting findings warrant further studies to ascertain the true host/s of PBVs, which still remains controversial. Until the true host/s of PBVs are proven, caution should be exercised during interpretation of PBV-related data in animals and humans, especially those on interspecies transmission events.

## Author Contributions

All authors listed have made a substantial, direct and intellectual contribution to the work, and approved it for publication.

## Conflict of Interest

The authors declare that the research was conducted in the absence of any commercial or financial relationships that could be construed as a potential conflict of interest.
